# Size-Dependent Internalization of Microplastics and Nanoplastics Using In Vitro Model of the Human Intestine—Contribution of Each Cell in the Tri-Culture Models

**DOI:** 10.3390/nano14171435

**Published:** 2024-09-02

**Authors:** Hyunjin Choi, Shohei Kaneko, Yusei Suzuki, Kosuke Inamura, Masaki Nishikawa, Yasuyuki Sakai

**Affiliations:** Department of Chemical System Engineering, Graduate School of Engineering, University of Tokyo, Tokyo 113-8656, Japansakaiyasu@chemsys.t.u-tokyo.ac.jp (Y.S.)

**Keywords:** microplastics, nanoplastics, intestine culture model, in vitro model, enterocyte, goblet cells, M cells, absorption pathway, size dependence, co-culture, tri-culture

## Abstract

Pollution by microplastics and nanoplastics (MNPs) raises concerns, not only regarding their environmental effects, but also their potential impact on human health by internalization via the small intestine. However, the detailed pathways of MNP internalization and their toxicities to the human intestine have not sufficiently been understood, thus, further investigations are required. This work aimed to understand the behavior of MNPs, using in vitro human intestine models, tri-culture models composed of enterocyte Caco-2 cells, goblet-like HT29-MTX-E12 cells, and microfold cells (M cells) induced by the lymphoblast cell line Raji B. Three sizes (50, 100, and 500 nm) of polystyrene (PS) particles were exposed as MNPs on the culture model, and size-dependent translocation of the MNPs and the contributions of each cell were clarified, emphasizing the significance of the tri-culture model. In addition, potential concerns of MNPs were suggested when they invaded the circulatory system of the human body.

## 1. Introduction

Plastics play an integral role in modern society, offering countless benefits to human life. In 2022, the estimated production amount of plastic around the world was 400.3 million tons, gradually increasing every year [1]. However, their poor degradability and limited recycling options have raised concerns regarding pollution from micro- and nanoplastics (MNPs) [2,3,4]. MNPs are tiny, fragmented plastic particles that result from the weathering [5,6], physical abrasion [7], and UV-induced photooxidation [8] of larger plastic items and are also artificially manufactured for industrial applications. They primarily consist of various plastic materials, such as polystyrene (PS), polypropylene (PP), polyethylene (PE), and polyethylene terephthalate (PET) [9,10]. MNPs can also be defined by their sizes; plastic particles smaller than 5 mm are referred to as microplastics, and further degraded microplastics smaller than 0.1 μm are specifically referred to as nanoplastics [11,12].

MNPs exist everywhere, in the air, ground, water, house dust, and a wide range of manufactured products, such as cosmetics, packaged beverages and food, and even in medical items [4,13]. One of the major concerns regarding MNP pollution is water, as water eventually serves as the primary pathway for oral exposure to MNPs in humans. Numerous studies have detected MNPs in various types of water, including ocean, surface water, groundwater, and even in tap water and bottled water that are closely linked to human life [14,15,16,17]. Furthermore, MNPs have already been detected in marine organisms used for seafood [7,16,17], providing evidence that MNP pollution in water can lead to human exposure to MNPs in the gastrointestinal tract (GIT), not only via drinking water, but also through the GIT. In 2016, the European Food Safety Agency (EFSA) reported the potential translocation of MNPs under 150 µm into the gut epithelium [18], and numerous in vivo studies (mainly rodents [19,20] and fish [21]) have already demonstrated MNP absorption in the GIT and accumulated in various organs. Moreover, many recent reports have detected MNPs in the human body, in stool, blood, lungs, placenta, and liver [13,22,23,24,25]. The exposure dose and the duration of MNP exposure are still up for debate; several in vivo and in vitro reports have suggested the toxicity of MNPs to the cells and organs by oxidative stress, inflammation, and cell death via apoptosis necrosis [19,26,27,28,29].

Absorption of MNPs in the GIT is considered to mainly occur in the small intestine, where nutrients and other substances are internalized [2,4,30]. Furthermore, the small intestine forms a protective barrier to shield the body from direct exposure to harmful substances, depending on the particle size and molecular characteristics [29,31,32,33,34,35]. Such selectivity in the small intestine is owing to the intrinsic function of the intestinal epithelial layer, which is composed of enterocytes, goblet cells, and microfold cells (M cells). The enterocyte forms a barrier with a tight junction (TJ) and regulates the uptake and transport of small molecules such as proteins, lipids, and other nutrients via paracellular and transcellular pathways to the blood capillaries [36,37,38,39]. The goblet cells secrete the mucus on the epithelium, forming a semi-permeable fibrous network membrane that suppresses the translocation of larger substances [40,41]. The M cells, located on follicle-associated epithelia (FAE), are specialized to uptake and transport macromolecules such as antigens and pathogens across to the lymphoid follicle on the basolateral of FAE, where many immune cells initiate immune responses [36,37,38,39]. However, knowledge of the correlations between MNPs and the selective transportation by the cell types is insufficient, and further studies on their interactions are considered necessary for a better understanding of MNP behavior in the GIT and their potential impact.

The enterocyte-like Caco-2 cells have been widely employed as an in vitro model of the intestinal epithelium for studies on the internalization and toxicity of nanoparticles for drug delivery and MNPs [29,31,39,42]. However, as Caco-2 cells lack mucus secretion and the capacity to uptake larger particles, this is thought to be a limit to representing the actual functionality of the intestinal epithelium. Thus, researchers have developed co- and tri-culture models that incorporate Caco-2 cells, HT29-MTX-E12 cells, and Raji B cells. HT29-MTX-E12 cells function as goblet-like cells capable of mucus secretion, and the Raji B cells induce the differentiation of Caco-2 cells into M cell-like cells [10,27,32,43]. Owing to these in vitro culture models, the behavior and toxicity of the MNPs on the intestinal epithelium are beginning to be unveiled, but there have been limited investigations into MNP studies using tri-culture models, and evaluations of MNP translocation remain insufficient. Moreover, the absence of standardized experimental methods results in variability across studies, such as cell types and exposure times, and MNP materials, sizes, and concentrations differ according to individual researchers. Consequently, the findings are fragmented, difficult to compare, and occasionally contradictory.

In this study, to elucidate the size-dependent translocation of MNPs and the contribution of each epithelial cell type in the GIT, we prepared in vitro monoculture, co-culture, and tri-culture models composed of Caco-2 cells and HT29-MTX-E12 cells, and M cells induced by Raji B cells. We selected commercially available polystyrene (PS) particles as MNPs, due to their accessibility and ease of handling, and since the inside of the particles are labeled with fluorescence, this allows for tracking their behavior and quantifying their amount using fluorescence. Three sizes (50, 100, 500 nm) of MNPs were exposed to culture models for 3 days, and their impacts on the barrier function of the epithelium and their behavior were evaluated.

## 2. Materials and Methods

### 2.1. Cell Culture

Colorectal adenocarcinoma epithelial cells (Caco-2), a human adenocarcinoma cell line with epithelial morphology, were obtained from RIKEN Bio Resource Center (Ibaraki, Japan). Caco-2 cells were cultured in Eagle’s Minimum Essential Medium (E-MEM, (Wako Pure Chemical Industries (Wako), Osaka, Japan)) supplemented with 20% fetal bovine serum (FBS), 1% non-essential amino acids (NEAA, Wako), and 1% penicillin–streptomycin (P.S., Wako). HT29-MTX-E12 cells, a mucus-secreting subclone from colon adenocarcinoma HT29 cells, differentiated into mature goblet cells by methotrexate, were obtained from the European Collection of Authenticated Cell Cultures-12040401 (ECACC, Salisbury, UK). HT29-MTX-E12 cells were cultured in low glucose Dulbecco Modified Eagle’s Medium (DMEM, Wako) supplemented with 10% FBS, 1% NEAA, and 1% P.S. Passage numbers between 42 and 53 were used for Caco-2 cells and between 30 and 45 were used for HT29-MTX-E12 cells. Raji B, a lymphoblast cell line, was obtained from the American Type Culture Collection (ATCC, Manassas, VA, USA). Raji B cells were cultured in Roswell Park Memorial Institute 1640 medium (RPMI-1640, Wako) supplemented with 10% FBS, 1% NEAA, and 1% P.S. These cells were maintained in a humidified atmosphere of 5% CO_2_ at 37 °C.

### 2.2. PS Particles

In this study, we used commercialized polystyrene (PS) spherical particles (Micromod, Rostock, German) with a plain surface as MNPs. Standard sizes of the particles were 50 nm, 100 nm, and 500 nm, and particles were labeled with a green fluorescent substance, which had a maximum excitation wavelength of 475 nm and a maximum fluorescence wavelength of 510 nm.

For the characterization of MNPs, the distribution of particle size and zeta potential of MNPs were verified by dynamic light scattering with a Zetasizer pro (Malvern Panalytical, Malvern, UK). MNPs were diluted in Milli-Q water (Millipore, Burlington, MA, USA) and high glucose-DMEM (HG-DMEM) with 10% FBS. The setting of sample temperature was maintained to 37 °C during the measurement. Additionally, to determine the stability of the fluorescence intensity of MNPs during the culture experiment, each of the MNPs with 0.1 mg/mL concentration were incubated in HG-DMEM containing 10% FBS for 3 days. The fluorescent intensity was measured with a NanoDrop 3300 Fluorospectrometer (Thermo Fisher Scientific, Waltham, MA, USA).

### 2.3. Culture Models for the MNP Exposure

**Figure 1:**
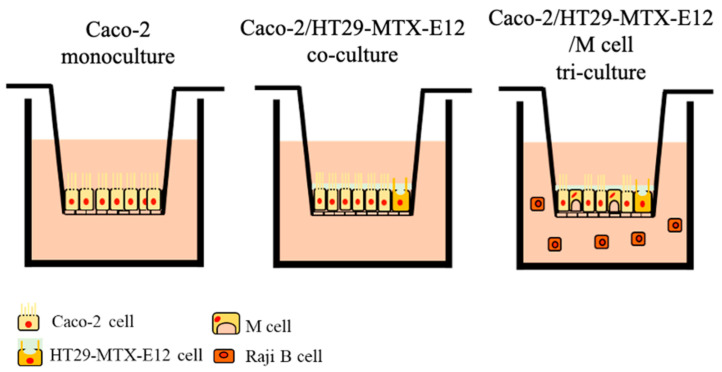
Schematic diagram of intestinal culture models. Monoculture of Caco-2 cells, co-culture of Caco-2 cells with HT29-MTX-E12 cells, tri-culture of Caco-2 cells with HT29-MTX-E12 cells and M cells induced by Raji B cells.

Caco-2 cells and HT29-MTX-E12 cells were seeded on the 24-well Transwell^®^ insert (pore size of 3.0 μm, growth area of 0.33 cm^2^, Corning, NY, USA) (Figure 1). Monoculture of Caco-2 cells and HT29-MTX-E12 cells were seeded 1 × 10^5^ cells/insert. In the co-culture, the ratio of Caco-2 cells to HT29-MTX-E12 cells at 9:1 was used for a total of 1 × 10^5^ cells/insert seeding. The medium was exchanged every 2 days. Cells were utilized for the MNPs exposure experiment after 18 days of culture. In the tri-culture, Caco-2 cells and HT29-MTX-E12 cells were seeded with the same method as co-culture and cultured for 14 days, and Raji B cells were seeded in the basolateral compartment of the culture insert at 1 × 10^6^ cells/well for the M cell induction, following previously described protocols [27,44]. Cells were cultured for 4 days more until day 18. The medium in both apical and basolateral compartments was exchanged every 2 days with a half volume, using HG-DMEM supplemented with 10% FBS, 1% NEAA, and 1% P.S. The prepared culture model was used for the MNP exposure; 0.1 mg/mL of 50 nm, 100 nm, and 500 nm MNPs were exposed for 72 h. The integrity of the epithelial cell layer and the translocation of MNPs were evaluated following the description below.

### 2.4. Evaluation of Epithelial Barrier Function: Transepithelial Electrical Resistance (TEER)

The integrity of the epithelial cell layer was determined by transepithelial electrical resistance (TEER) using an epithelial volt-ohm meter equipped with a chopstick electrode (Millicell ERS-2, Millipore). The TEER value was monitored at each time point.

### 2.5. Measurement of MNP Translocation

To determine the permeability of MNPs across the epithelial cell layer, the particle concentration of the medium in the apical compartment and basolateral compartment of the Transwell^®^ insert were measured using a NanoDrop 3300 Fluorospectrometer. The number of permeated MNPs were calculated based on the concentration of permeated MNPs and their standard size.

### 2.6. Immunofluorescent Staining

After MNP exposure, exposed cells were washed with phosphate buffer solution (PBS). For zonula occludens-1 (ZO-1), Mucin2 (MUC2), and Glycoprotein 2 (GP2) staining, 4% paraformaldehyde phosphate buffer solution (Wako) was used to fix the cells. Next, the cells were blocked using 1% bovine serum albumin (BSA) in PBS. The cells were incubated with primary antibodies overnight at 4 °C and, subsequently, with secondary antibodies for 60 min at 25 °C. The primary antibody targets used were ZO-1 (human anti-ZO-1 rabbit antibody, abcam, Cambridge, UK), MUC2 (human anti-MUC2 mouse antibody, abcam, UK), and GP2 (human anti-GP2 mouse antibody, Abnova, Taipei, Taiwan), and antibodies were diluted at 1:100~1:200. The secondary antibodies were goat anti-rabbit IgG Alexa Fluor 568 (1: 1000, Thermo Fisher Scientific) and goat anti-mouse IgG Alexa Fluor 647 (1: 1000, Thermo Fisher Scientific). Then, the cells were incubated with 0.2 µg/mL 4′,6-diamidino−2-phenylindole (DAPI) (Dojindo Molecular Technologies, Inc., Kumamoto, Japan) for 10 min to visualize nuclei. After the bottom of the Transwell^®^ with the cell layer was excised and placed on a thin glass plate, the stained cells were observed with confocal laser microscopy (FV3000, Olympus, Tokyo, Japan). Fluorescence observation of nuclei with the 405 nm laser, green fluorescent-modified MNPs with the 488 nm laser, and target markers with the 561 and 640 nm lasers, including xy-layer and Z stacks, was performed to obtain images.

### 2.7. Statistical Analysis

All data were expressed as mean ±  standard deviation (SD) from independent experiments. Data were statistically analyzed using Student’s *t*-test. Values of *p* < 0.01 or 0.05 were considered to represent a statistically significant difference. Differences between sample groups were analyzed using multiple-way analysis of variance (ANOVA) followed by Tukey’s post hoc test. The *p* value of <0.01 or 0.05 was considered significant.

## 3. Results

### 3.1. The Characterization of MNPs

**Figure 2:**
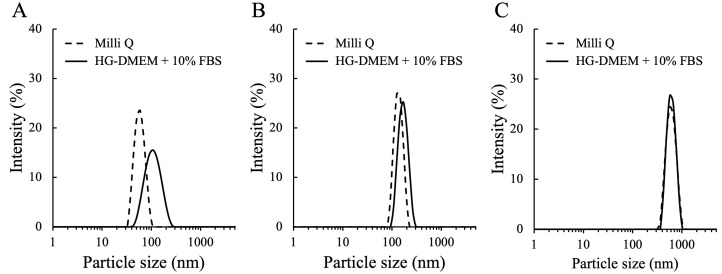
DLS curves for the (**A**) 50 nm MNP, (**B**) 100 nm MNP, (**C**) 500 nm MNP, diluted in the Milli Q water and HG-DMEM with 10% FBS.

To understand the characteristics of MNPs used in this work, the hydrodynamic size and the zeta potential of MNPs were determined by DLS (Figure 2) (Table 1). In the Milli Q water, MNPs showed approximate size to the standard size, and the low polydispersity index (PDI) indicated MNPs have a homogenous size distribution. On the other hand, the size of MNPs were larger in the medium with FBS. This result can be explained by the absorption of proteins (mainly the bovine serum albumin, the most abundant protein in FBS) to the MNP surface to form protein corona that modify the characteristic of particles [45]. Especially, the effect from the protein was thought to be greater to smaller MNPs in this work. The 50 nm and 100 nm MNPs were inferred to be prone to aggregate, while the 500 nm MNPs did not increase much. The results of zeta potential showed the surface charge of each MNP is neutral in the medium with FBS (Table 1).

To confirm the fluorescence stability of MNPs, each size MNP was incubated with DMEM high glucose medium containing FBS for 3 days, and their fluorescence intensities were measured after incubation (Supplementary Figure S1). The fluorescence intensities of the 50 nm and 100 nm MNPs did not decrease significantly, while 500 nm MNPs decreased by approximately 20%. Consequently, the quantification of 500 nm MNPs in this study can be underestimated, but it is not thought to be so critical and is sufficiently stable for quantitative analysis.

### 3.2. Impact of MNP Exposure on Caco-2 Cell Monoculture: Barrier Integrity and Behavior of MNPs during Exposure

**Figure 3:**
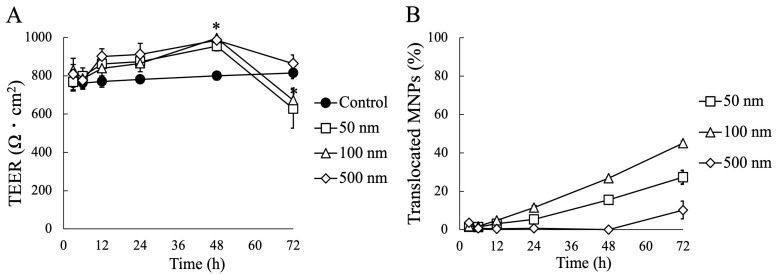
Effect of MNP exposure on Caco-2 monoculture. (**A**) TEER measurement for the evaluation of barrier integrity, (**B**) percentage of translocated MNPs across the Caco-2 cell layer. Data shown represent the mean ± SD (n = 6) from three independent experiments, (* *p* < 0.01).

To assess the impact of MNPs on the barrier function of Caco-2 cells, we monitored the daily changes in the TEER values of the Caco-2 cell layer (Figure 3A). The results demonstrated that the MNPs significantly affected the integrity of the Caco-2 monolayer. All sizes of MNPs led to an increase in TEER values up to 48 h, followed by a decrease at 72 h. Notably, the 50 nm and 100 nm MNPs exhibited a significant reduction in TEER values compared to the control after 72 h of exposure, which indicated that the potential toxicity of MNPs on Caco-2 cells and smaller MNPs such as 50 nm and 100 nm MNPs is more critical than 500 nm MNPs.

The permeability of MNPs across the Caco-2 cell layer was assessed by quantifying the percentage of translocated MNPs (Figure 3B). After 72 h, approximately 27.3% of the 50 nm MNPs and 45.1% of the 100 nm MNPs had translocated, whereas only 10.2% of the 500 nm MNPs successfully crossed the cell layer. These results suggest that 50 nm and 100 nm MNPs have a significantly higher translocation efficiency through the Caco-2 cell layer compared to 500 nm MNPs.

**Figure 4:**
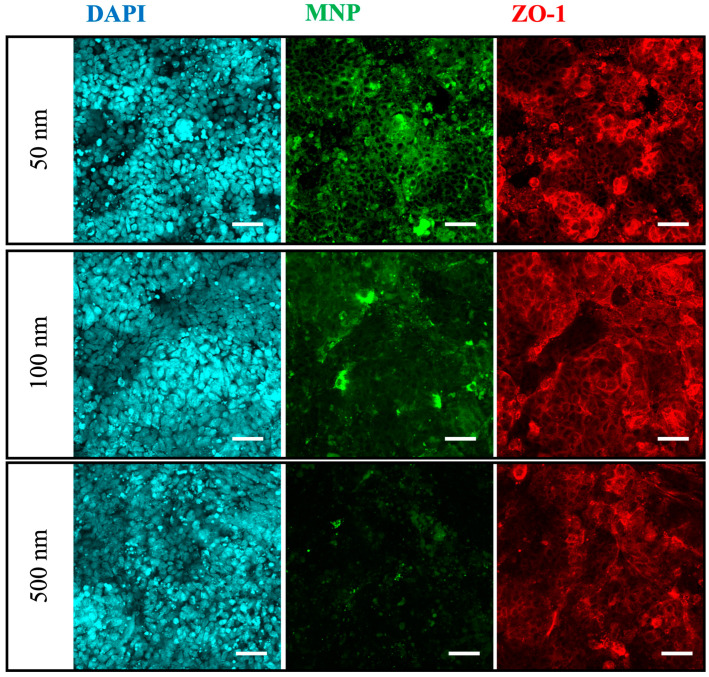
Confocal microscopy was conducted to observe the localization of exposed MNPs in the Caco-2 monoculture. Cyan: DAPI, Green: MNPs, Red: ZO-1. Scale bars = 50 µm.

We performed confocal microscopy to confirm the localization of MNPs on the Caco-2 monolayer, and we observed MNPs were absorbed onto the cell surface (Figure 4). Smaller MNPs covered a larger area of the cell surface. However, there were no significant deformations of the TJ protein ZO-1 structure under any of the conditions. These results suggest that MNPs can absorb into the cell surface, but there was no significant damage on the TJ formation, even with the TEER decrease after 72 h of MNP exposure.

### 3.3. Impact of MNP Exposure on Co- and Tri-Culture of Intestinal Epithelium

**Figure 5:**
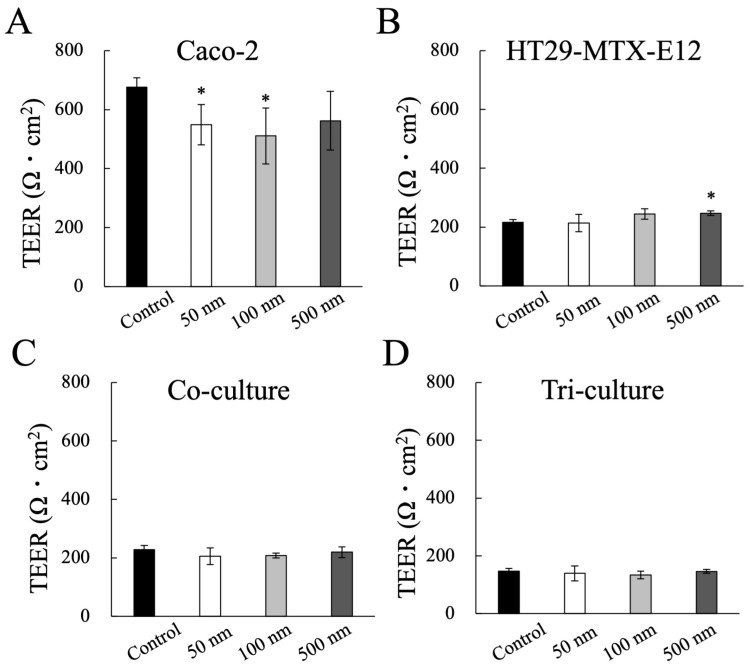
Effect of MNP exposure on Caco-2 cells barrier integrity. (**A**) Caco-2 monoculture, (**B**) HT29-MTX-E12 monoculture, (**C**) co-culture of Caco-2 with HT29-MTX-E12, (**D**) tri-culture of Caco-2 with HT29-MTX-E12 and M cells. Data shown represent the mean ± SD (n = 6) from three independent experiments, (* *p* < 0.01).

Following the investigations of MNP exposure on the monoculture of Caco-2 cells, we newly conducted MNP exposure on the monoculture of Caco-2 cells and HT29-MTX-E12 cells, using co- and tri-culture systems comprising three types of intestinal epithelial cells for enhanced physiological properties. Consistent with previous reports, the TEER values in the HT29-MTX-E12 monoculture, co-, and tri- culture were downregulated compared to the Caco-2 monoculture, due to the reduced formation of TJ by HT29-MTX-E12 cells and M cells (Figure 5) [46,47]. However, the culture conditions with HT29-MTX-E12 cells showed no decrease in the TEER value after MNP exposure, while the TEER value decreased in the Caco-2 monoculture with 50 nm and 100 nm MNPs. These results suggest that goblet cells can minimize the impact of MNPs on the barrier function of the intestinal epithelium.

### 3.4. Translocation and Localization of MNPs in Co- and Tri-Culture of Intestinal Epithelium

**Figure 6:**
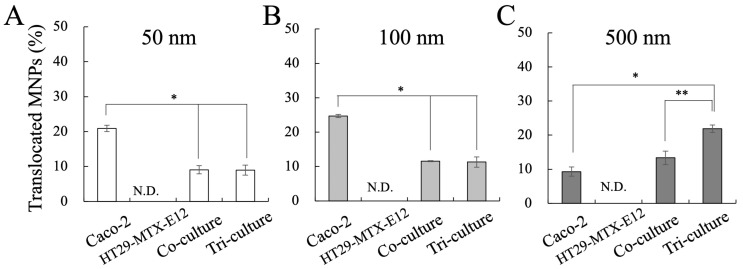
Translocation of MNPs in each culture condition. (**A**) MNP 50 nm, (**B**) MNP 100 nm, (**C**) MNP 500 nm. Data shown represent the mean ± SD (n = 6) from three independent experiments, (* *p* < 0.01, ** *p* < 0.05).

**Figure 7:**
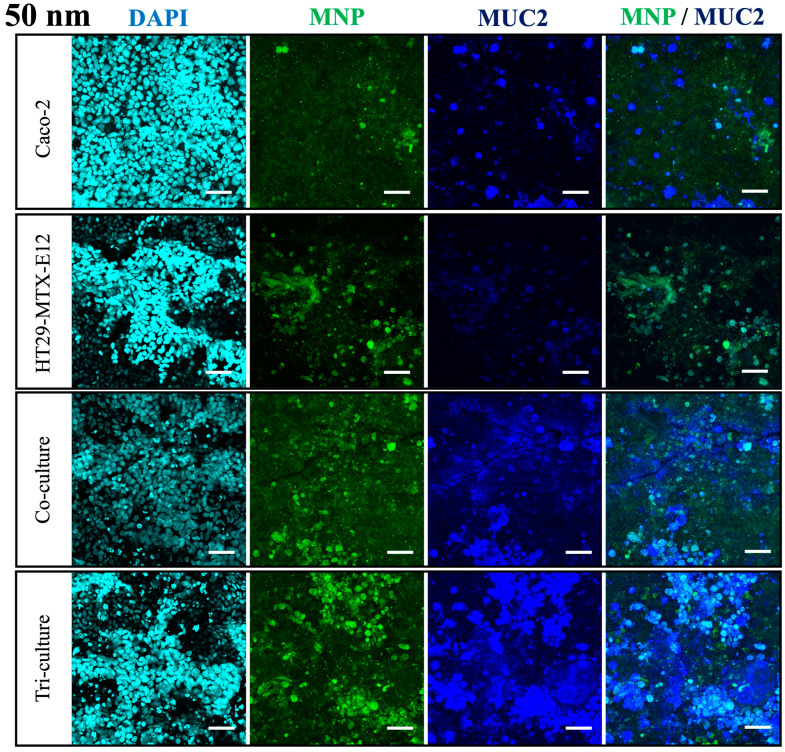
Observation of MUC2 expression and localization of 50 nm MNPs in each culture condition using confocal microscopy. Cyan: DAPI, Green: MNPs, Blue: MUC2. Scale bars = 50 µm.

From the permeability measurements, we observed the translocation of MNPs in the Caco-2 monoculture, co-culture, and tri-culture, whereas the monoculture of HT29-MTX-E12 did not exhibit MNP translocation (Figure 6). Furthermore, translocation of 50 nm and 100 nm MNPs were inhibited by HT29-MTX-E12 cells, suggesting the mucus disturbs the penetration of particles. Interestingly, the permeability of 500 nm MNPs was not affected much by HT29-MTX-E12 cells in this result, which conflicts with previous findings, since the permeability in the mucus is known to decrease with larger particle size [48]. On the other hand, from the confocal microscopy, we observed significant expression of mucus protein MUC2 in the culture with HT29-MTX-E12 cells, and MNPs were localized on the MUC2 (Figure 7). These results indicate the protective role of the HT29-MTX-E12 cells, producing mucus to capture the MNPs to inhibit the translocation.

**Figure 8:**
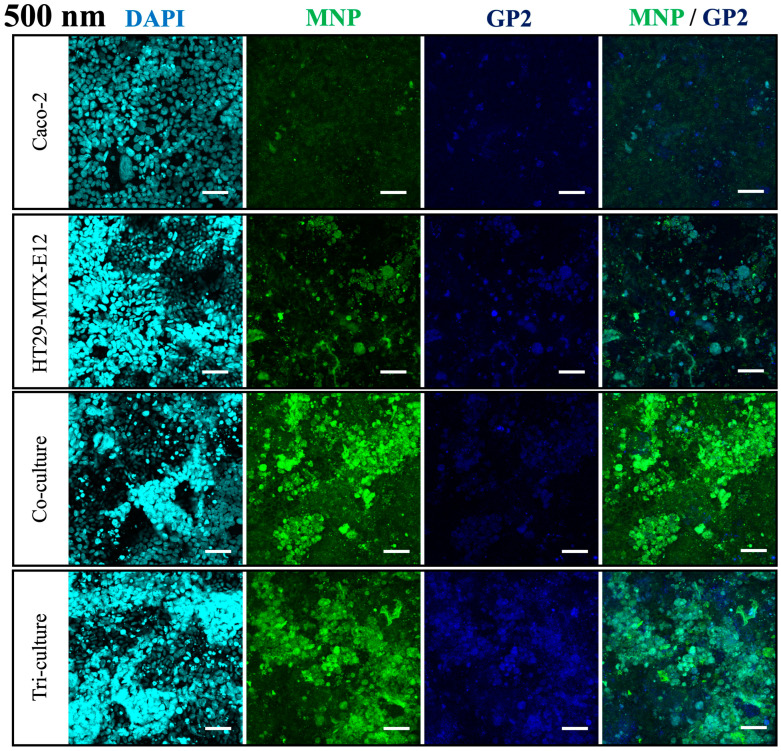
Observation of GP2 expression and localization of 500 nm MNPs in each culture condition using confocal microscopy. Cyan: DAPI, Green: MNPs, Blue: GP2. Scale bars = 50 µm.

Moreover, the permeability of 500 nm MNPs was significantly increased in the tri-culture incorporating M cells (Figure 6C) when compared to the Caco-2 monoculture and co-culture, while the translocation of 50 nm and 100 nm MNPs remained unaffected under tri-culture (Figure 6A,B). Overall, considering the results from the Caco-2 monoculture, the translocation of 50 nm and 100 nm MNPs is considered to mainly depend on Caco-2 cells, and larger 500 nm MNPs mainly depend on M cells. Moreover, the confocal microscopy revealed the expression of GP2 in the tri-culture, which is a marker of M cells, proving the M cell induction is successful, and the overlay of GP2 expression with 500 nm MNP localization suggested the uptake of 500 nm by M cells (Figure 8). On the other hand, in the basolateral compartment of the tri-culture, where the Raji B lymphocyte cell line was inoculated for M cell induction, we observed that the translocated MNPs were taken up by the Raji B cells (Supplementary Figure S2). This suggests that in the human body, MNPs transported by M cells could be re-uptaked by immune cells, as the basolateral side of M cells is rich in immune cells.

In addition, from the calculations on the number of translocated MNPs based on the translocated MNP concentration (Figure 6) and their standard size, smaller MNPs showed more dominant translocation counts even with less translocation percentage (Supplementary Figure S3), suggesting that the smaller MNPs have a much greater chance to translocate to the human body.

## 4. Discussion

The increasing awareness of the presence and potential threats posed by MNPs in our environment has raised concerns about their impact on human health, and many researchers are trying to understand the behavior of MNPs in the intestinal epithelium in relation to their size to predict the influence of MNPs on the human body. Owing to in vitro culture models using intestinal epithelial cells in monoculture, co-culture, and tri-culture systems, the approximate behaviors of MNP translocation across the intestinal epithelium are being unveiled. Nonetheless, due to the lack of comprehensive comparative analysis across mono-, co-, and tri-culture systems, and variations in experimental conditions, the findings vary, leading to a complex and conflicting understanding.

In this study, we conducted MNP exposure with 3 sizes, 50 nm, 100 nm, and 500 nm, on Caco-2 cells or HT29-MTX-E12 cell monocultures, and co-cultures of Caco-2 cells and HT29-MTX-E12 cells, and tri-cultures of Caco-2 cells and HT29-MTX-E12 cells with M cells, to evaluate the translocation and localization of MNPs and their impact on barrier function. This comprehensive comparative approach aims to minimize the knowledge gap on the behavior and impact of MNPs on the intestinal epithelium.

According to the results from the MNP exposure on the intestinal epithelium culture models with mono-, co-, and triculture, we confirmed the role of each cell, enterocyte, goblet cells, and M cells. Enterocytes contributed to the selective translocation of small MNPs (50, 100 nm), goblet cells contributed to the protection of the epithelium against MNP translocation by secreting mucus, and M cells contributed to the translocation of large MNPs (500 nm). The enterocyte-like cells, Caco-2 showed efficient translocation of 50 and 100 nm MNPs, while 500 nm MNPs showed lower permeability. This result is supported by previous knowledge that enterocyte-mediated translocation of particles occurs predominantly for smaller than 1 μm size, and smaller particles have higher transport rates [43,49]. On the other hand, the translocation of 500 nm MNPs was enhanced by the tri-culture with M cells. The M cell is characterized by the specialized internalization of macromolecules such as antigens and microbes, and several in vitro studies proved that particles over 500 nm to 10 μm are translocated by M cells [10,37]. In addition, in the culture with goblet-like cells HT29-MTX-E12, we confirmed HT29-MTX-E12 cells secrete mucus, which is known to form a semi-permeable membrane on the intestinal epithelial layer [50], serving as a barrier to limit the penetration of MNP. HT29-MTX-E12 cells did not facilitate the MNP translocation, rather they captured MNPs in the mucus and efficiently reduced the translocation of 50 and 100 nm MNPs, a consequence of preventing cell damage on the cell layer. Overall, these findings indicate that each cell contributes to size-dependent translocation of MNPs and protection of the intestinal epithelium from MNPs.

The impact of internalized MNPs was expected to affect different circulatory systems depending on their particle size. Small MNPs translocated by enterocyte are considered to invade blood capillaries connected to the blood circulatory system, and subsequently, get passed to the liver, which works as a filter in the circulatory system against foreign substances [36,37,38,39]. Furthermore, larger MNPs translocated by M cells are considered to be transported to lymphoid follicles on the basolateral of M cells, which contain immune cells, such as macrophages, dendritic cells, and lymphocytes [36,37,38,39]. In particular, macrophages are considered to actively uptake large particles up to 10 μm, even though they could not eliminate MNPs with their enzymes in lysosomes [10,51,52]. Therefore, for the additional investigations on internalized MNPs, the effect of small MNPs on the liver and the effect of larger MNPs on immune cells, particularly macrophages, are thought to be suitable options for the next steps.

In this study, we adapted the culture models of Caco-2 cells, HT29-MTX-E12 cells, and Raji B cells due to their robustness and accessibility. However, these cells, derived from cancerous tissues, exhibit abnormalities that raise questions about their representativeness of the human intestinal epithelium. For example, Caco-2 cells demonstrate abnormally high TEER values with limited permeability, while HT29-MTX-E12 cells exhibit a lack of mucus secretion. Especially, the thickness of the mucus layer secreted by HT29-MTX-E12 cells is known to be 3–5 μm [50], while the mucus layer found in the small intestine of humans is reported to be 450–900 μm thick [40,53]; thus, the influence of MNP in the actual human body will be different from in vitro culture systems. One conflict resulting from this work is that the permeability of 500 nm MNPs was not impaired with HT29-MTX-E12 cells (Figure 6), whereas previous findings demonstrated that larger particles have lower permeability in the mucus. There is some possibility that the 3–5 μm of mucus is not thick enough to inhibit large particles, leading to the unexpected results.

To address these issues concerning cell sources, several researchers have explored the development of intestinal epithelial cells derived from organoids. They produced enterocyte-like, mucus-secreting goblet-like cells from organoids, as well as M cell-like cells, inducing with the nuclear factor-kappa B ligand (RANKL) [33,54,55]. Recently, Ying Chen et al. reported that they applied organoid-derived intestinal culture models to MNP investigations, demonstrating the M cell functions, and found toxicity of 30 and 100 nm MNPs in high concentrations over 0.5 mg/mL. However, despite these advancements, there are still missing parts involving evaluations of mucus secretion and quantification of MNP translocation across the cell layer [33]. Thus, it is thought that additional investigations are required to understand MNP behavior in culture models with higher physiology.

In this work, we adapted commercialized polystyrene (PS) particles as MNPs due to their ease of accessibility and handling. These particles have round shape with a hard and plain surface. However, as these properties of PS particles cannot fully represent actual MNPs in an environment with various types of materials, sizes, and surfaces, the nanomechanical cues (stiffness, hardness, elasticity, viscosity) [51,52] that can affect the absorption of MNPs are not well described in this work. Thus, for future work, the mechanical properties of MNPs should be considered.

Moreover, there is a need for culture models designed to study the translocation of larger MPs, specifically those exceeding 10 μm. MPs up to 130 μm are considered able to translocate across the intestinal epithelium [18,30]. An in vivo study with mice demonstrated that 20 μm PS particles were found in the liver and kidneys after oral administration [20], and the size range of 4–30 μm plastics was found in human liver tissue in cirrhotic conditions [23]. However, the pathways and mechanisms of larger MP translocation are not well understood because these findings cannot be explained by M cells, as the maximum transport of M cells is known to be 10 μm. Furthermore, the translocation studies of MPs larger than 10 μm have been constrained due to the physical limitations imposed by the pore size of standard Transwell systems, which typically have a maximum pore size of 8 μm, and the larger MPs cannot permeate. Therefore, to improve our understanding of the larger MPs internalization in the intestine, the development of novel in vitro culture models that can permeate larger MPs is required.

For the evaluation of MNP toxicity on the intestinal epithelium, it was thought that a long-term study is required for precise predictions of MNP toxicity. We assessed the barrier integrity using TEER measurements, and the potential toxicity of MNPs on the epithelium was suggested. In the Caco-2 monoculture experiment, TEER values initially showed a significant temporary increase for 2 days following MNP exposure but decreased by day 3. The reason for this temporal exaltation of barrier integrity with MNPs remains unclear in this work. However, given that the barrier integrity of epithelial cells is known to react sensitively to external stimuli from the chemicals, microbes, and nanoparticles [56,57,58], we hypothesize that the MNPs may have stressed the cells, prompting an increase in tight junction formation as an initial defensive response before the toxicity manifested [57]. However, despite a decrease following MNP exposure, the barrier integrity of the Caco-2 monoculture remained intact, with no significant abnormalities observed in ZO-1 formation. Additionally, the downregulation of TEER values was effectively suppressed by mucus-secreting cells. Moreover, numerous previous studies have reported no acute toxicity from MNP exposure, except at high concentrations exceeding 0.5 mg/mL [10,28,33,59]. However, given the observed trend, it is possible that the decrease in TEER values in the Caco-2 monoculture was ongoing on day 3, suggesting that prolonged MNP exposure could lead to further cell damage. These findings indicate the need for extended exposure experiments with MNPs to gain a more comprehensive understanding.

## 5. Conclusions

In this study, aiming to bridge the knowledge gaps concerning the internalization behaviors of nano- and microplastics in the intestinal epithelium, we conducted a comprehensive analysis using three sizes (50, 100, 500 nm) of MNPs in mono-, co-, and tri-cultures of Caco-2 cells, HT29-MTX-E12 cells, and M cells, which represent complex epithelial functions. From overall comparisons, we confirmed the size-dependent MNP translocations belonging to the cell types, the translocation of 50–100 nm MNPs depends on enterocytes, and over 500 nm MNPs depends on M cells; these results support the previous knowledge. Furthermore, we emphasized the importance of the protective role of mucus that can efficiently minimize the internalization and adverse effects of MNPs. In addition, we suggested several points of improvement in the investigations for more precise predictions, long-term studies with better physiological culture models, and further investigations on the MNPs after they have invaded the human body, which will be helpful for better understanding. In conclusion, we believe our work will contribute to a better understanding of MNP behavior in the small intestine and will be a clue for future investigations.

## Figures and Tables

**Table 1:** Particle size and zeta potential of MNPs determined by DLS.

MNP	Dispersant	Size (nm)	Zeta Potential (mV)	PDI
50 nm	Milli Q	58.76 ± 1.37	−34.65 ± 1.20	0.04 ± 0.01
	HG-DMEM + 10% FBS	113.40 ± 2.80	−8.32 ± 1.47	0.17 ± 0.01
100 nm	Milli Q	139.00 ± 2.64	−24.72 ± 2.74	0.02 ± 0.03
	HG-DMEM + 10% FBS	171.40 ± 0.48	−9.32 ± 0.74	0.02 ± 0.01
500 nm	Milli Q	603.50 ± 9.57	−6.87 ± 0.09	0.05 ± 0.03
	HG-DMEM + 10% FBS	625.10 ± 10.79	−10.23 ± 1.25	0.04 ± 0.03

## Data Availability

The raw data supporting the conclusions of this article will be made available by the authors on request.

## Supplementary Materials

The following Supporting Information can be downloaded at: https://www.mdpi.com/article/10.3390/nano14171435/s1, Figure S1: Stability of the fluorescent intensity of MNPs after 3 days in HG-DMEM with 10% FBS, Figure S2: Uptake of MNPs by Raji B cells in the basolateral compartment of tri-culture, Figure S3: Number of translocated MNPs.
