# Supplementary material for: Size-Dependent Internalization of Microplastics and Nanoplastics Using In Vitro Model of the Human Intestine—Contribution of Each Cell in the Tri-Culture Models
Source: Nanomaterials (Basel). 2024 Sep 2;14(17):1435. doi: 10.3390/nano14171435 (PMC11397364; doi:10.3390/nano14171435)
Supplement: Supplementary file 1 [file nanomaterials-14-01435-s001.zip › nanomaterials-3168617-supplementary.pptx]

## Slide 1
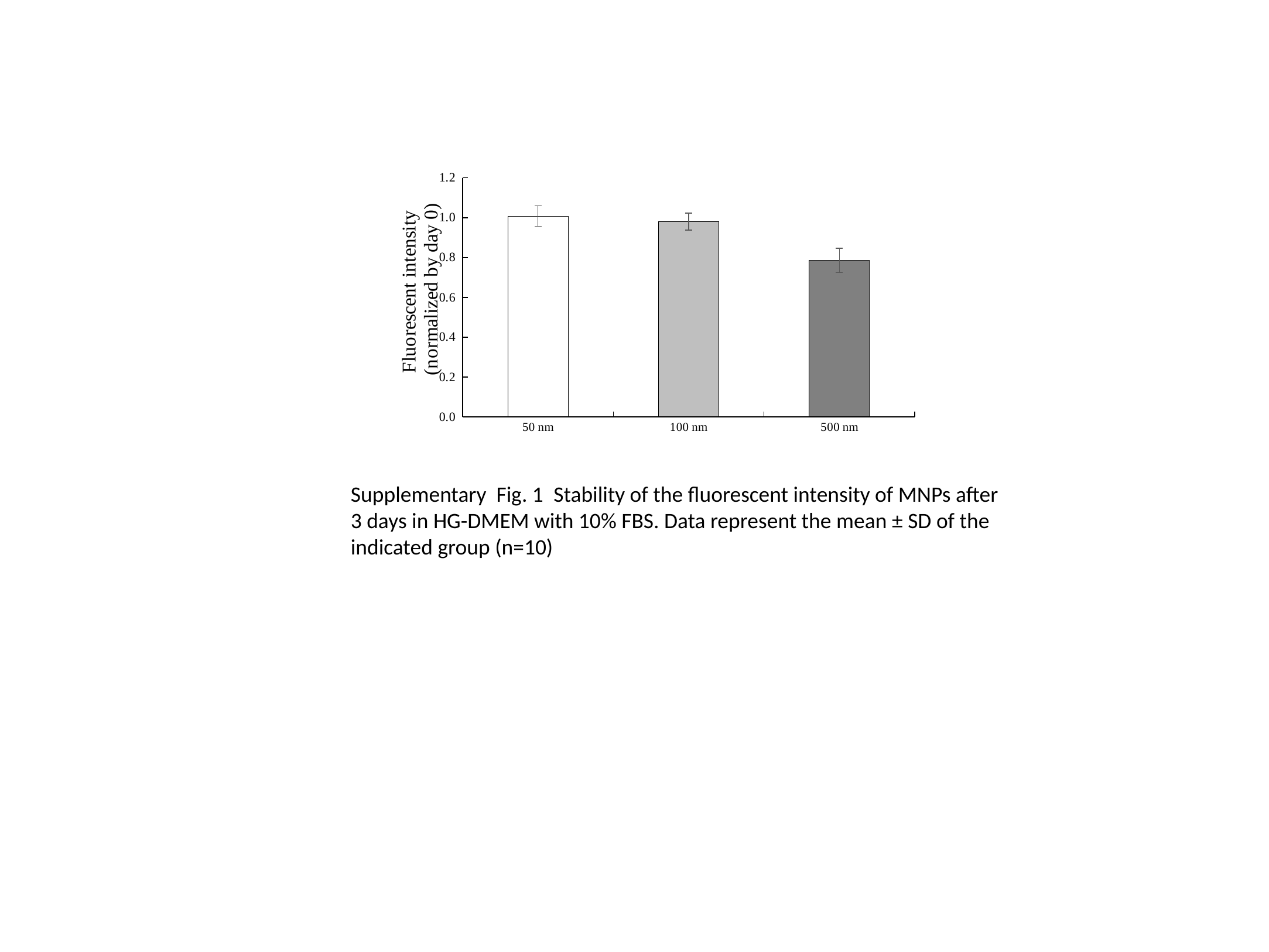

### Chart
| Category | |
|---|---|
| 50 nm | 1.008046293186466 |
| 100 nm | 0.9800624727486478 |
| 500 nm | 0.7854281082320618 |Supplementary Fig. 1 Stability of the fluorescent intensity of MNPs after 3 days in HG-DMEM with 10% FBS. Data represent the mean ± SD of the indicated group (n=10)

## Slide 2
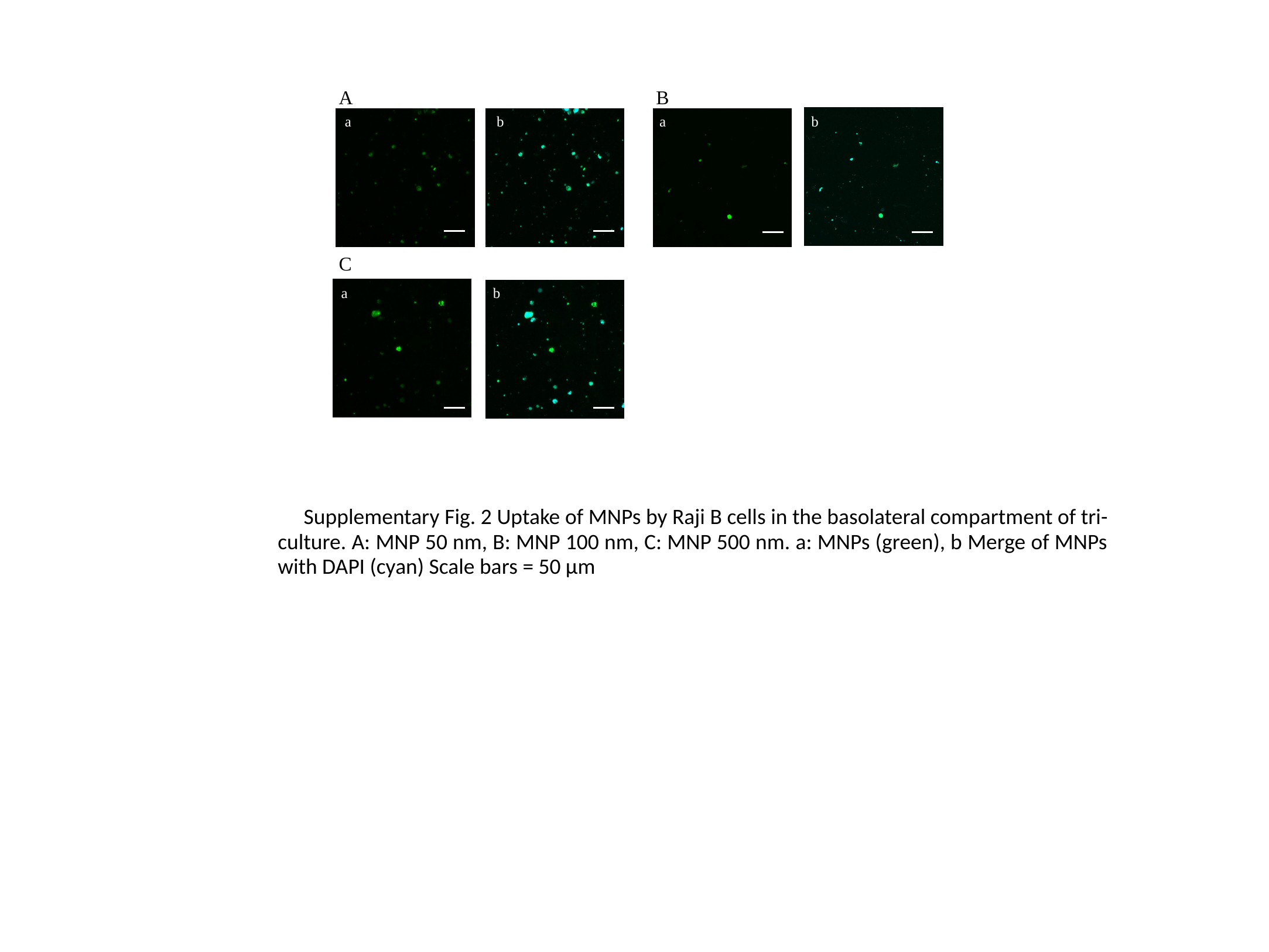

A
B
b
b
a
a
C
b
a
Supplementary Fig. 2 Uptake of MNPs by Raji B cells in the basolateral compartment of tri-culture. A: MNP 50 nm, B: MNP 100 nm, C: MNP 500 nm. a: MNPs (green), b Merge of MNPs with DAPI (cyan) Scale bars = 50 µm

## Slide 3
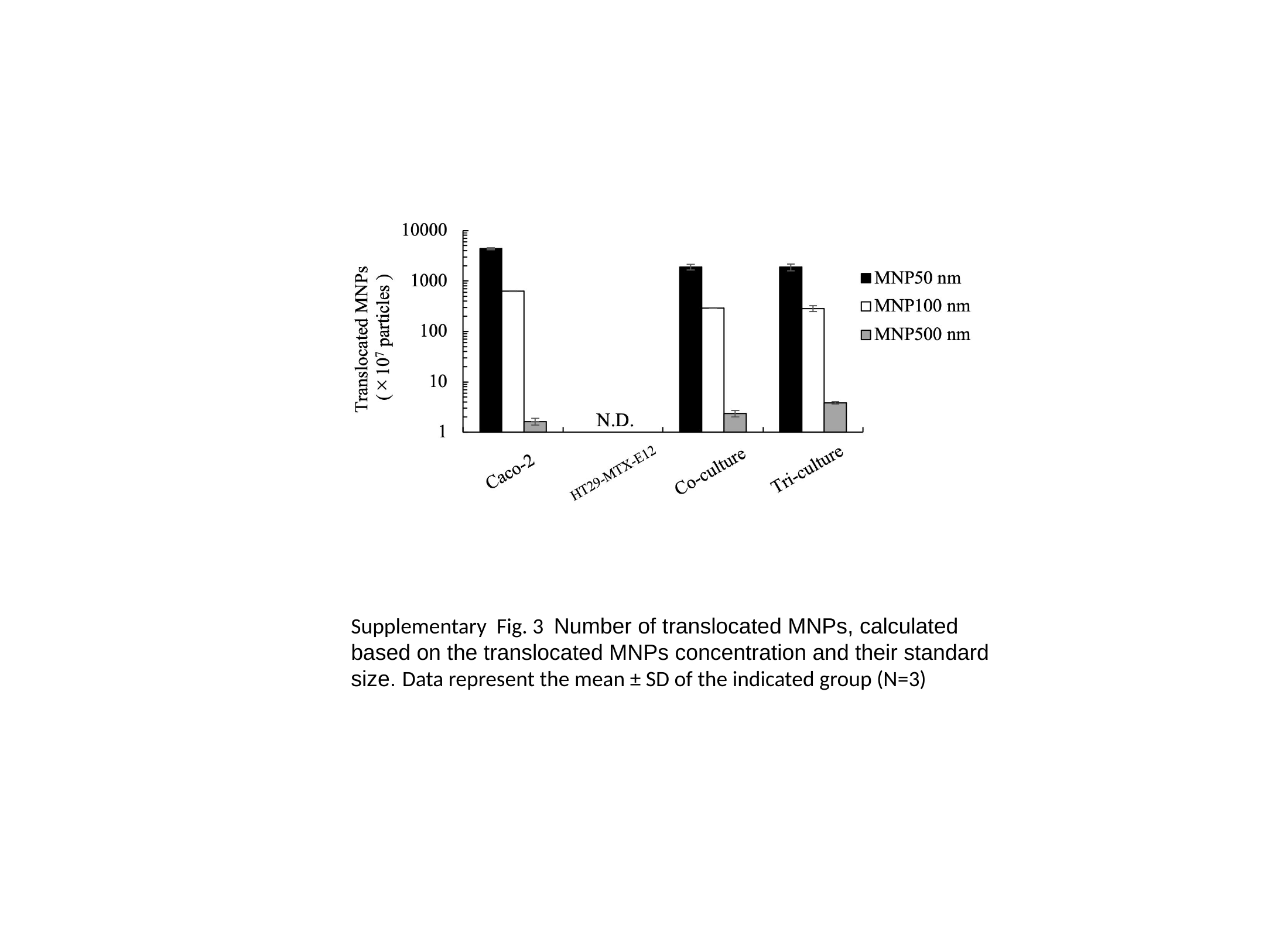

Supplementary Fig. 3 Number of translocated MNPs, calculated based on the translocated MNPs concentration and their standard size. Data represent the mean ± SD of the indicated group (N=3)
